# Effect of airflow angle on abaxial surface deposition in air-assisted spraying

**DOI:** 10.3389/fpls.2023.1211104

**Published:** 2023-07-04

**Authors:** Shaoqing Xu, Xiang Wang, Chao Li, Xiangkai Ran, Yuan Zhong, Ye Jin, Jianli Song

**Affiliations:** ^1^College of Science, China Agricultural University, Beijing, China; ^2^Centre for Chemicals Application Technology, China Agricultural University, Beijing, China; ^3^Sanya Institute of China Agricultural University, Sanya, China; ^4^Weichai Lovol Intelligent Agricultural Technology Co., Ltd., Shandong, Weifang, China

**Keywords:** air-assisted spray, abaxial surface, coverage, angle of airflow, exposure

## Abstract

Air-assisted sprayers are widely used in orchards for pest and disease control. However, air-assisted spray deposition on the abaxial surface of leaves is often limited. In this study, a method to achieve satisfactory spray deposition on the abaxial leaf surface and an assessment of factors that affect abaxial surface deposition were investigated. The effects of leaf angle, wind speed, platform velocity, and nozzle type were assessed. Abaxial surface coverage was significantly affected by leaf angle, wind speed, and nozzle type, of which the leaf angle had the strongest impact. The leaf angle largely determines the abaxial surface area exposed to the wind field. When the abaxial surface is situated leeward, deposition of droplets on the abaxial surface is difficult. Therefore, to improve abaxial surface exposure for field application, the exposure probability of the abaxial surface at different angles between the leaf and the airflow (α) was examined. The relationship was well represented by a logistic growth curve. The exposure probability exceeded 95% when the α value was greater than 5°. The latter finding was verified by conducting a field application in which the deposition efficiency on the abaxial surface (DEAS) was calculated. Adjustment of the airflow angle based on the theoretical value achieved DEAS of 49.9% and 109.3% in the middle and upper layers of the canopy, respectively, whereas the DEAS was less than 30% if the airflow angle was not adjusted. This is caused by the difference in the exposure probability of the back of the leaf. The results provide a reference for adjustment of the wind field of air-assisted sprayers in field applications.

## 1 Introduction

Air-assisted spraying is an efficient ground-based spray application technology recommended by the Food and Agriculture Organization of the United Nations (FAO, 2001). The auxiliary airflow may cause the leaves to turn over or oscillate. The spray droplets are directed by the airflow to penetrate the canopy, which improves the uniformity of spray distribution within the canopy (Burgio et al., 2016; Zhou et al., 2016). In addition, disruption from natural wind flow and droplet drift are reduced by using a suitable airflow rate, and the pesticide utilization can be improved (Zhu et al., 2004).

For most types of fruit trees, the abaxial leaf surface has a greater number of stomata and a thinner cuticle than the adaxial surface (Toselli et al., 2009; Carr, 2013; Carr, 2014), and is the main site of pathogen infection (Washington et al., 1998; Churchill, 2011). Pests, such as red spider mites and whitefly, also tend to be more frequent on the abaxial leaf surface (Laurence et al., 1978). Therefore, adequate deposition of pesticides on the abaxial surface of leaves is required for effective pest control in fruit trees. However, deposition on the abaxial surface is often inadequate or uneven in air-assisted spraying. In addition, adjustment of the spray volume has little effect on deposition on the abaxial surface (Garcerá et al., 2020).

Airflow is another important factor affecting deposition. The state of leaves is changed when the airflow changes (Zhang et al., 2022). The reconfiguration and vibration of the leaf vary under different airflow characteristics (Jiang et al., 2021). Deposition can be affected by aerodynamic response speed (Li et al., 2021). Field application also showed that the adjustment of airflow influences droplet deposition between the medial and lateral parts of the canopy (Svensson et al., 2003; Pai et al., 2009). A computational fluid dynamics (CFD) simulation revealed that deposition on the abaxial leaf surface was strongly associated with the airflow angle (Wang et al., 2015). Therefore, airflow direction is an important factor that must be considered. In most orchard air-assisted sprayers, wind direction is varied mainly by adjusting the guide plate. A strong correlation between leaf droplet deposition in the vertical profile of the canopy and sprayer airflow direction has been reported (Duga et al., 2015). Furthermore, improved deposition is achieved by adjusting the angle of the air outlet (Celen, 2008; Pai et al., 2009; Grella et al., 2022).

Recommendation manuals and devices have been developed that allow rough adjustment of the airflow characteristics (TOPPS-Prowadis Project, 2014; Garcerá et al., 2017). However, these adjustments need to be based on specific canopy characteristics, such as canopy size and leaf density. Hence, the recommendations are not readily implemented in practical applications. In addition, there is currently a lack of guideline data for the improvement of deposition on the abaxial leaf surface during application. Therefore, in this study, factors that influence droplet deposition on the abaxial leaf surface were investigated. The effect of the angle between the leaf and airflow on exposure of the abaxial surface was examined, and the optimal angle was calculated. The findings provide a reference for the adjustment of air-assisted sprayers in practical applications.

## 2 Methods

### 2.1 Effect of parameters setting on deposition on the abaxial surface

**Figure 1:**
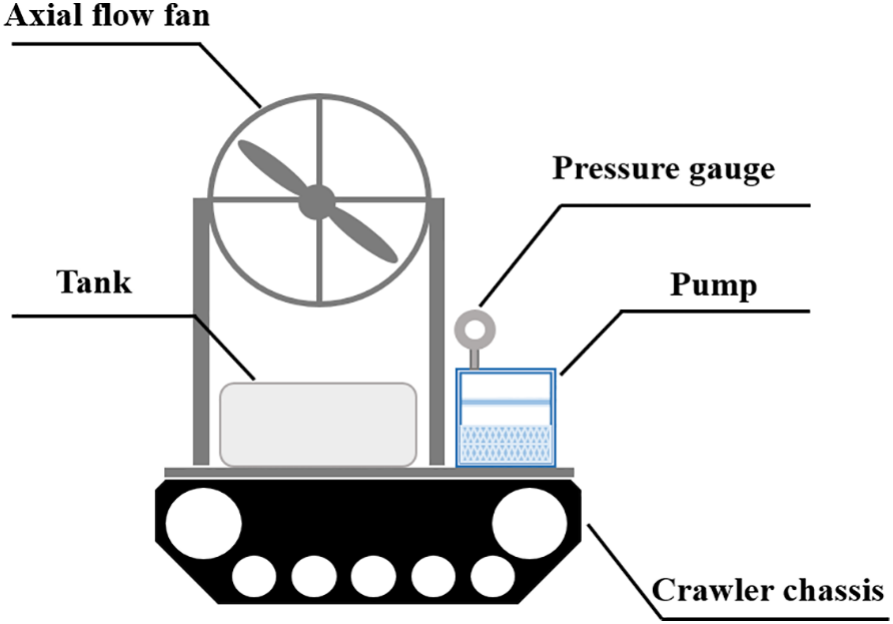
Schematic illustration of the movable spray platform used in the initial experiment.

A movable spray platform was used in the initial experiment (**Figure 1**). The platform was composed of an air-assisted spraying system and a crawler chassis. The air-assisted sprayer system comprised a 24 V power source, an axial fan (blade diameter 38 cm), a motor, a speed regulator, a pressure gauge, a centrifugal pump, and a nozzle. The motor speed was controlled by a speed regulator with an adjustable range from 1200 to 3600 rpm. The small tracked chassis was operated by remote control for constant speed movement.

**Figure 2:**
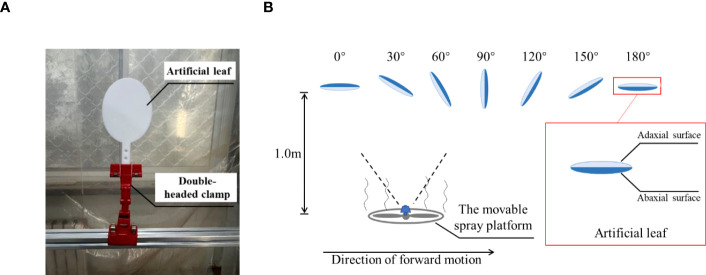
Experimental setup for spray deposition on the abaxial surface of an artificial leaf. **(A)** Method of securing the position of an artificial leaf. **(B)** The tested angles of an artificial leaf relative to the direction of forward motion.

An artificial leaf was used, which was made from a polyvinyl chloride sheet of 5 mm thickness. Unlike a real leaf, the artificial leaf would not deform in the wind field. Therefore, the angle between the artificial leaf and the direction of forward motion of the movable spray platform was fixed during the experiment. A double-headed clamp was used to fix the artificial leaf in position (**Figure 2A**). The artificial leaf was fixed at the same height as the center of the axial fan. The head of the clamp could be rotated to alter the angle between the leaf surface and the direction of forward motion. Seven artificial leaves were fixed on the test frame. The distance between each leaf was 0.2 m. The tested angles between the leaf surface and the direction of forward motion of the movable spray platform were 0°, 30°, 60°, 90°, 120°, 150°, and 180° (**Figure 2B**). Water-sensitive paper (WSP) was secured to the abaxial surface of the artificial leaf.

The horizontal distance between the axial fan and the artificial leaf was 1.0 m. The experimental parameters in each treatment group are summarized in **Table 1**. The rotation speed was 2700 or 3600 rpm. The forward speed of the movable spray platform was 0.8 or 1.2 m s^−1^. The nozzle types used in the experiment were TR80-02 and TR80-005C hollow-cone nozzles (Lechler, Düsseldorf, Germany). The spray pressure was 3.0 bar. After spraying, the WSP was removed and scanned at 600 dpi resolution. Deposit Scan (National Institutes of Health, Bethesda, MD, USA) was used to determine the spray coverage for each artificial leaf. Each treatment group comprised three replications.

**Table 1:** Parameters for spray deposition on the abaxial surface of the artificial leaf.

Treatment group	Rotation speed of the fan (rpm)	Platform velocity (m s^−1^)	Outlet wind speed (m s^−1^)	Nozzle type
1	2700	0.8	8	TR80-02
2	3600	0.8	12	TR80-02
3	2700	1.2	8	TR80-02
4	3600	1.2	12	TR80-02
5	3600	1.2	12	TR80-005C

### 2.2 Effect of airflow angle on exposure of the abaxial surface

In this experiment, the probability of exposure of the abaxial surface to the airflow was evaluated. In addition, the minimum angle between the leaf abaxial surface and the airflow for effective deposition was calculated. The leaf used in the experiment was from a citrus tree located at the China Agricultural University. To ensure the freshness of the leaves, the experiment was conducted within 10 min of their collection. The fan used was identical to that described in section 2.1. The direction of the axial fan was parallel to the ground.

**Figure 3:**
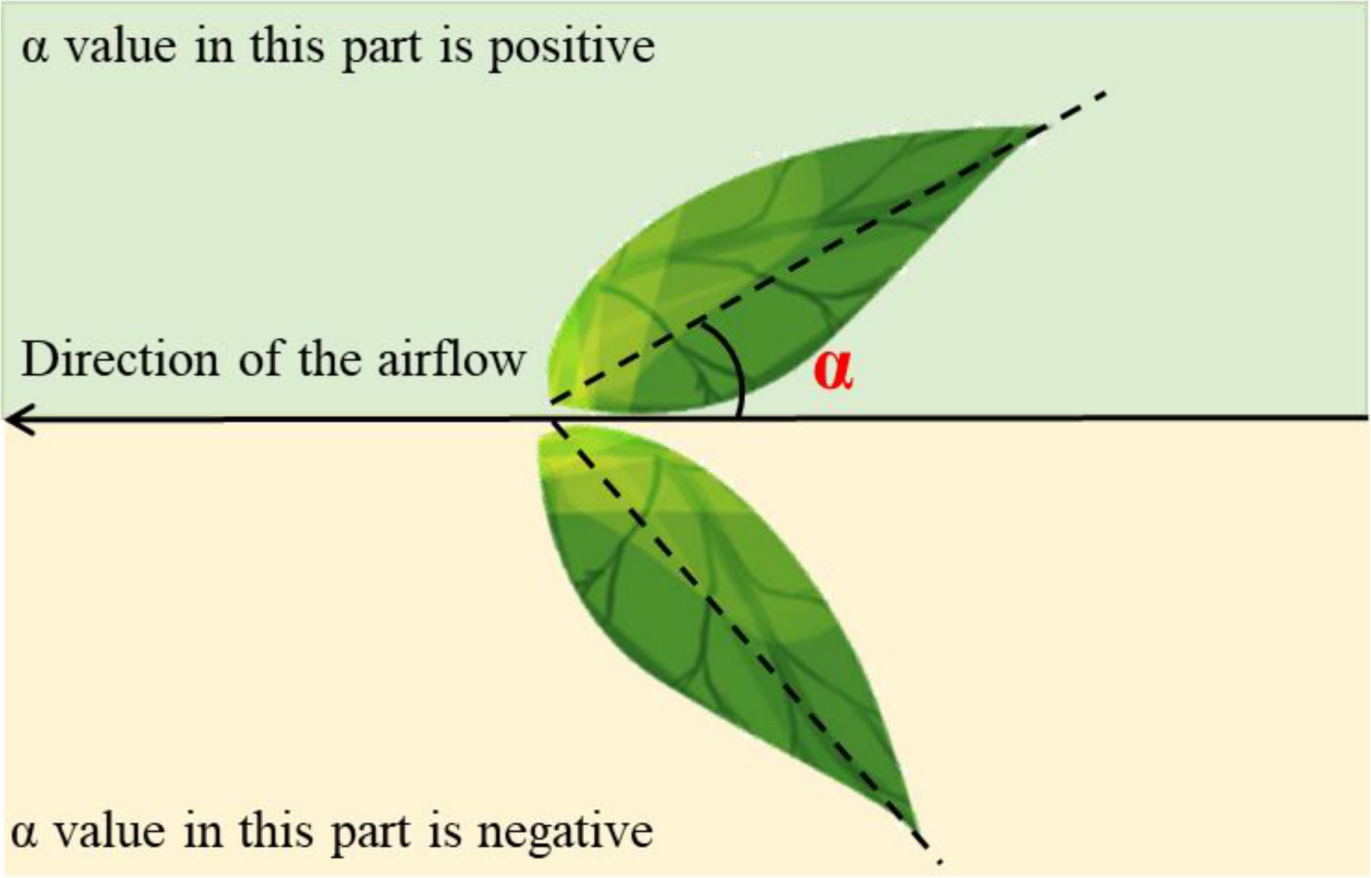
Schematic diagram illustrating the angle α between a leaf and the airflow direction.

The angle α between the leaf and the airflow direction was varied during the experiment (**Figure 3**). The α value was adjusted at 5° intervals from −40° to 40°. The leaf was fixed to an iron rod with clamps during the experiment. The leaf inclination angle was adjusted to the preset value with the aid of an angle-measuring instrument (ROK International Industry Co., Ltd., Shenzhen, China). The air-assisted spray platform used was identical to that described in section 2.1. The rotation speed was set to 3600 rpm. The fan was maintained at the same height as the center of the leaf. The horizontal distance between the fan and the leaf was 1.0 m. The velocity of the platform was 1.2 m s^−1^. A Gopro Hero 7 digital camera (Gopro, Inc., San Mateo, CA, USA) was used to record the oscillation of the leaf. Thirty repetitions were performed for each α value.

**Figure 4:**
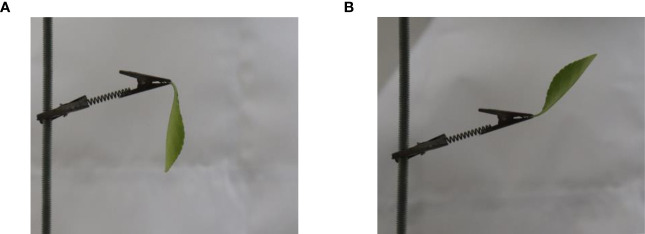
Two states of a leaf in the wind field. **(A)** The abaxial surface of the leaf is not exposed to the wind field. **(B)** The abaxial surface of the leaf is exposed to the wind field.


The leaf oscillation in the recorded video was observed and scored. If the abaxial surface was exposed to the wind field (**Figure 4B**), the result was recorded as 1; if the abaxial surface was not exposed to the wind field (**Figure 4A**), it was recorded as 0. The probability of abaxial surface exposure to the wind field was calculated for each α. The probability (as the dependent variable on the *y*-axis) for each α (as the independent variable on the *x*-axis) was plotted. In addition, a growth curve function (Equation 1) was fitted to the data:

(1) $$y=\frac{a}{1+e^{-k\left(x-x_{c}\right)}}$$

where *x* is the angle between the fan and the leaf, *y* is the probability of abaxial surface exposure, and *a*, *k*, and *x*_c_ are constants.

### 2.3 Spray coverage with different airflow angles in field application

A field experiment was conducted to verify the applicability of the findings of the experiment described in section 2.2. The spraying equipment used was basically identical to the movable spray platform described in section 2.1. The difference was that the number of spray units was increased to three. Each spray unit was fixed to the connecting rod by adjustable fasteners at an angle adjustable from −20° to 20°. The distance between the center of adjacent fans was 0.5 m.

The experiment was conducted in a citrus orchard greenhouse in the Xiao Tangshan Agricultural Demonstration Park, Beijing, China. The spacing between rows of citrus trees was 2.8 m. The average height of the trees was 1.7 m. Three trees were selected for the experiment. The leaf angles in the upper, middle, and lower vertical layers of the canopy were measured. Thirty leaves in each layer were measured. The leaf angle was defined as the angle between the abaxial surface and the horizontal plane. The mean leaf angles were 16.5° in the upper layer, −10.2° in the middle layer, and −43° in the lower layer. The angles of the fans were adjusted according to the conclusions from the experiment described in section 2.2 (**Table 2**). However, the angle of the lower fan in treatment group 1 could not be adjusted to the preset value because of the structure of the fan. Therefore, an angle of 20° was used. Fans in the control (treatment group 2) were set parallel to the horizontal plane.

**Table 2:** Airflow angles applied in the field experiment.

Treatment group	Parameter	Canopy layer		
		Upper	Middle	Lower
1	Airflow angle (°)	−6.5	20.2	20
	α (°)	10	10	−23
2	Airflow angle (°)	0	0	0
	α (°)	16.5	−10.2	−43

The angle of the axial fan was adjusted relative to the horizontal plane (0°) to generate the specified airflow angle. α is the mean angle between the abaxial surface of a leaf (n = 30) and the airflow direction. Treatment group 2 (airflow angle = 0°) served as the control.

**Figure 5:**
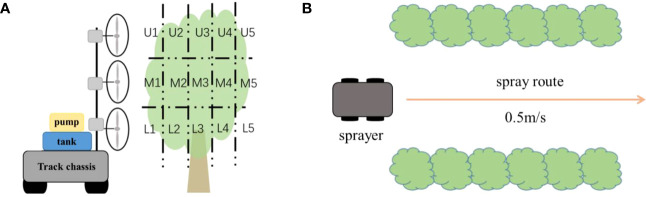
Schematic diagram of the experimental setup for the field application. **(A)** Division of the canopy of a citrus tree into horizontal and vertical layers. **(B)** The route used in the spray application.

Three citrus trees located on the spraying route were selected. The canopy of each tree was divided into 15 areas for measurement of spray distribution (**Figure 5A**) in accordance with the guidelines in ISO 22522:2007. The canopy was divided into three layers (upper [U], middle [M], and lower [L]) in the vertical profile and five sectors sequentially along the horizontal airflow direction (numbered 1 to 5). The layer nearest to the axial fan was termed the first layer and the layer farthest from the fan was the fifth layer. In each of the 15 areas of the canopy, three leaves were selected and WSP was secured to the adaxial and abaxial surfaces of each leaf. The distance between the sprayer and the tree row was 1.5 m. The nozzle used in the experiment was a TR80-02 hollow-cone nozzle (Lechler). The pressure was 5 bar and the velocity of the sprayer was 0.5 m s^−1^. The rotation speed was 3600 rpm. To observe the effect of the airflow angle on spray deposition onto the leaf surfaces, only one side of the tree row was sprayed (**Figure 5B**). After spraying, the WSPs were collected and scanned at 600 dpi resolution. Deposit Scan was used to determine the spray coverage in each area of the canopy. Each treatment was repeated three times.

The deposition efficiency on the abaxial surface (DEAS) was used to express the ability for droplet deposition on the abaxial surface of the leaf, which was calculated using Equation 2:


(2) $$DEAS\;\left(\%\right)=\frac{ABSC}{ADSC}\times 100$$

where ABSC is spray coverage on the abaxial surface and ADSC is spray coverage on the adaxial surface.

Given that the airflow angle may affect droplet penetration, the penetration rate in each area of the canopy was calculated using Equation 3:


(3) $$Penetration\;rate\;\left(\%\right)=\;\frac{C_{i}}{C_{1}}\times 100$$

where *C*_1_ is the spray coverage of the first layer and *C_i_* is the spray coverage of layer *i*.

## 3 Results

### 3.1 Effect of parameters setting on spray deposition on the abaxial surface of an artificial leaf

The spray coverage on the abaxial surface of an artificial leaf, as influenced by four application parameters, is summarized in **Table 3**. The angle between the artificial leaf and the direction of forward motion of the movable spray platform significantly affected the coverage. At an angle between 0° and 90°, spray coverage on the abaxial surface was less than 2% in all treatments. In these cases, the abaxial surface was situated leeward, making droplet deposition difficult. Nevertheless, a small number of droplets were deposited on the abaxial surface. At an angle exceeding 90°, a significant increase in spray coverage on the abaxial surface was observed. In group 2, for example, the coverage was 1.6%, 18.7%, 34.1%, and 49.8% at 90°, 120°, 150°, and 180°, respectively, and at these angles, the differences in coverage were statistically significant (*P* ≤ 0.05). Similar patterns were observed for the other treatments. The abaxial surface was directly exposed to the wind field at an angle greater than 90° and the degree of exposure increased gradually as the angle increased. These results indicated that deposition under air-assisted spraying was positively correlated with the degree of exposure to the wind field.

**Table 3:** Spray coverage on the abaxial surface of an artificial leaf at different included angles.

Treatment group	Included angle (°)						
	0	30	60	90	120	150	180
1	0.9 ± 0.3 c	0.2 ± 0.1 c	0.4 ± 0.1 c	1.2 ± 0.6 c	10.3 ± 3.9 b	17.2 ± 5.4 ab	21.0 ± 6.6 a
2	1.0 ± 0.8 d	0.5 ± 0.1 d	0.3 ± 0.3 d	1.6 ± 0.8 d	18.7 ± 11.2 c	34.1 ± 13.2 b	49.8 ± 5.5 a
3	0.1 ± 0.1 d	0.2 ± 0.1 d	0.1 ± 0.1 d	0.6 ± 0.5 d	8.3 ± 3.1 c	16.5 ± 3.7 b	29.3 ± 2.9 a
4	0.2 ± 0.1 d	0.3 ± 0.2 d	0.1 ± 0.0 d	0.8 ± 0.2 d	19.0 ± 9.3 c	28.9 ± 5.0 ab	27.9 ± 4.9 a
5	0.1 ± 0.0 b	0.3 ± 0.2 b	0.1 ± 0.0 b	0.5 ± 0.4 b	8.0 ± 2.3 a	7.7 ± 0.7 a	11.9 ± 4.4 a

Values are the mean ± standard deviation (n = 3). Different lowercase letters within a column indicate a significant difference between means (one-way ANOVA; p< 0.05).

The effects of four application parameters on spray coverage were considered. Multi-factor ANOVA was used to analyze the effects of the included angle, the rotation speed of the fan, platform velocity, and nozzle type on spray coverage on the abaxial surface (**Table 4**). A significant effect was observed for the included angle, rotation speed of the fan, and nozzle type (*P* ≤ 0.05). The included angle was observed to have the strongest significant effect on spray coverage (*F*-value = 54.8).

**Table 4:** Significance of the effects of application parameters on spray coverage on the abaxial surface of an artificial leaf.

Parameter	df	*F*	Significance
Included angle	6	54.8	*
Rotation speed of the fan	1	24.2	*
Platform velocity	1	2.6	−
Nozzle type	1	19.4	*

− P > 0.05, * P ≤ 0.05.

### 3.2 Effect of airflow angle on exposure of the abaxial surface

**Figure 6:**
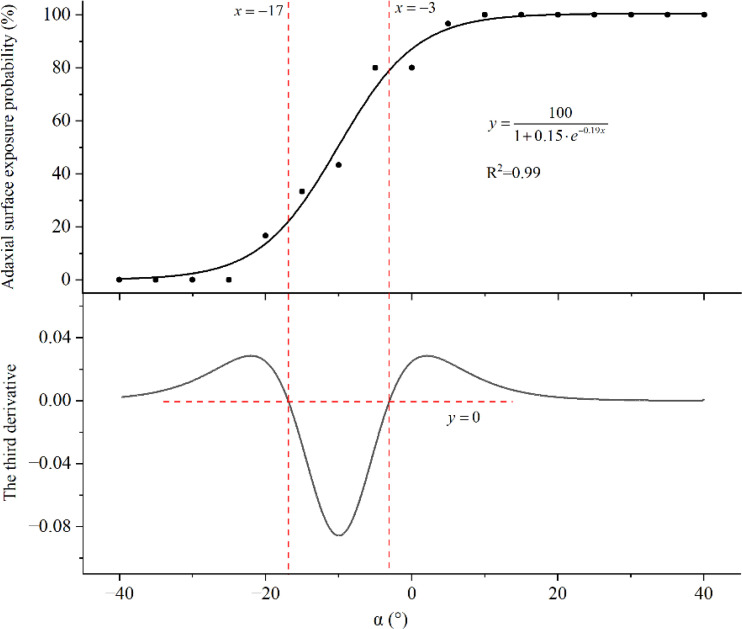
Fitted growth curve and the third-order derivative for the probability of exposure of the leaf’s abaxial surface.

The probability of exposure of the leaf’s abaxial surface under different α values is summarized in **Table 5**. At α< −25°, the abaxial surface was not exposed to spray droplets. With an increase in α, the degree of exposure gradually increased. At α ≥ 10°, the abaxial surface was entirely exposed to the wind field. The growth curve fitted to the data as well as the third-order derivative is shown in **Figure 6**. The curve was divided into three stages based on the positive and negative values of the third-order derivatives: α ≤ −17° (first stage), −17°< α ≤ −3° (second stage), and α > −3° (third stage). In the first stage, the leaves were located too low in the vertical profile of the canopy and were forced downwards by the wind field. Thus, the abaxial surface’s probability of exposure was low (less than 22%). In the second stage, with an increase in α, the probability of exposure increased rapidly. At α > −3°, the rate of increase in the probability of exposure declined. In this stage, the probability of abaxial surface exposure was high (greater than 80%). Thus, it was observed that the abaxial surface had a high probability of exposure to the airflow at an airflow angle slightly less than the leaf angle. The probability of abaxial surface exposure at α = 10° was greater than 98%. Therefore, an α value of 10° was chosen for the following field experiment.

**Table 5:** Probability of exposure of the abaxial leaf surface to spray deposition at different airflow angles (α).

α (°)	−40	−35	−30	−25	−20	−15	−10	−5	0	5	10	15	20	25	30	35	40
Number of leaves with deposition on abaxial surface	0	0	0	0	5	10	13	24	24	29	30	30	30	30	30	30	30
Exposure probability (%)	0	0	0	0	17	33	43	80	80	97	100	100	100	100	100	100	100

### 3.3 Comparison of coverage under different α values

Spray coverage in the different canopy areas for the two treatments is summarized in **Table 6**. Paired-sample *t*-tests were performed to assess the treatment effects on spray coverage in the same canopy area. For the leaf adaxial surface, no significant differences were observed in all areas. However, for the abaxial surface, significant differences were observed in the middle layer (*P* ≤ 0.05). Based on the conclusion from the experiment described in section 3.2, α affects the abaxial surface’s probability of exposure. In the middle layer, α was 10° in group 1, which led to a high probability of abaxial surface exposure. In contrast, the α value in group 2 was −10.2° and the probability of abaxial surface exposure was less than 50%. This accounted for the difference in spray coverage on the abaxial surface between the two treatments. In addition, for the upper layer, α was always greater than 10° for the two treatments. Therefore, high spray coverage was consistently observed, with an average coverage of 10.3% and 16.7% in groups 1 and 2, respectively. For the lower layer, α was less than −20° in two treatments, which resulted in low spray coverage.

**Table 6:** Spray coverage on the adaxial and abaxial leaf surfaces in different canopy areas for the two treatments.

Canopy area		Adaxial surface of leaves				Abaxial surface of leaves			
		Group 1		Group 2		Group 1		Group 2	
Upper canopy	U1	14.4 ± 10.8	a	9.7 ± 6.9	a	16.7 ± 11.4	a	25.8 ± 11.2	a
	U2	10.3 ± 7.3		6.8 ± 3.7		17.0 ± 8.7		28.1 ± 6.7	
	U3	10.8 ± 6.7		8.3 ± 6.1		8.1 ± 9.9		22 ± 21.3	
	U4	6.8 ± 4.8		6.6 ± 4.6		7.4 ± 5.3		5.8 ± 6.2	
	U5	4.7 ± 2.4		6.8 ± 2.7		2.2 ± 2.8		2.0 ± 2.7	
	Average	9.4		7.6		10.3		16.7	
Middle canopy	M1	21.1 ± 12.8	a	23.2 ± 9.6	a	8.6 ± 8.8	a	6.7 ± 9.3	b
	M2	23.4 ± 10.4		18.6 ± 11.3		7.9 ± 3.5		4.7 ± 3.1	
	M3	4.8 ± 3.3		11.5 ± 10.5		6.9 ± 7.4		3.8 ± 6.8	
	M4	2.2 ± 1.5		2.5 ± 1.7		1.8 ± 2.5		1.9 ± 2.2	
	M5	2.4 ± 2.4		3.1 ± 1.6		1.7 ± 3.9		0.3 ± 0.3	
	Average	10.8		11.8		5.4		3.5	
Lower canopy	L1	23.5 ± 11.8	a	30.3 ± 12.2	a	3.8 ± 3.6	a	1.8 ± 1.4	a
	L2	19.7 ± 9.6		30.6 ± 8.6		3.2 ± 3.7		6 ± 8.3	
	L3	13.1 ± 7.1		6.7 ± 6.7		6.1 ± 5.8		6.5 ± 6.8	
	L4	12.5 ± 9.8		5.6 ± 5.1		3.3 ± 4.7		1.4 ± 1.0	
	L5	3.0 ± 1.6		2.2 ± 1.5		1.5 ± 2.3		2.0 ± 2.3	
	Average	14.4		15.1		3.6		3.6	

Values are the mean ± standard deviation (n = 3). Different lowercase letters within a column indicate a significant difference between means (pared-samples T test; p< 0.05).

**Figure 7:**
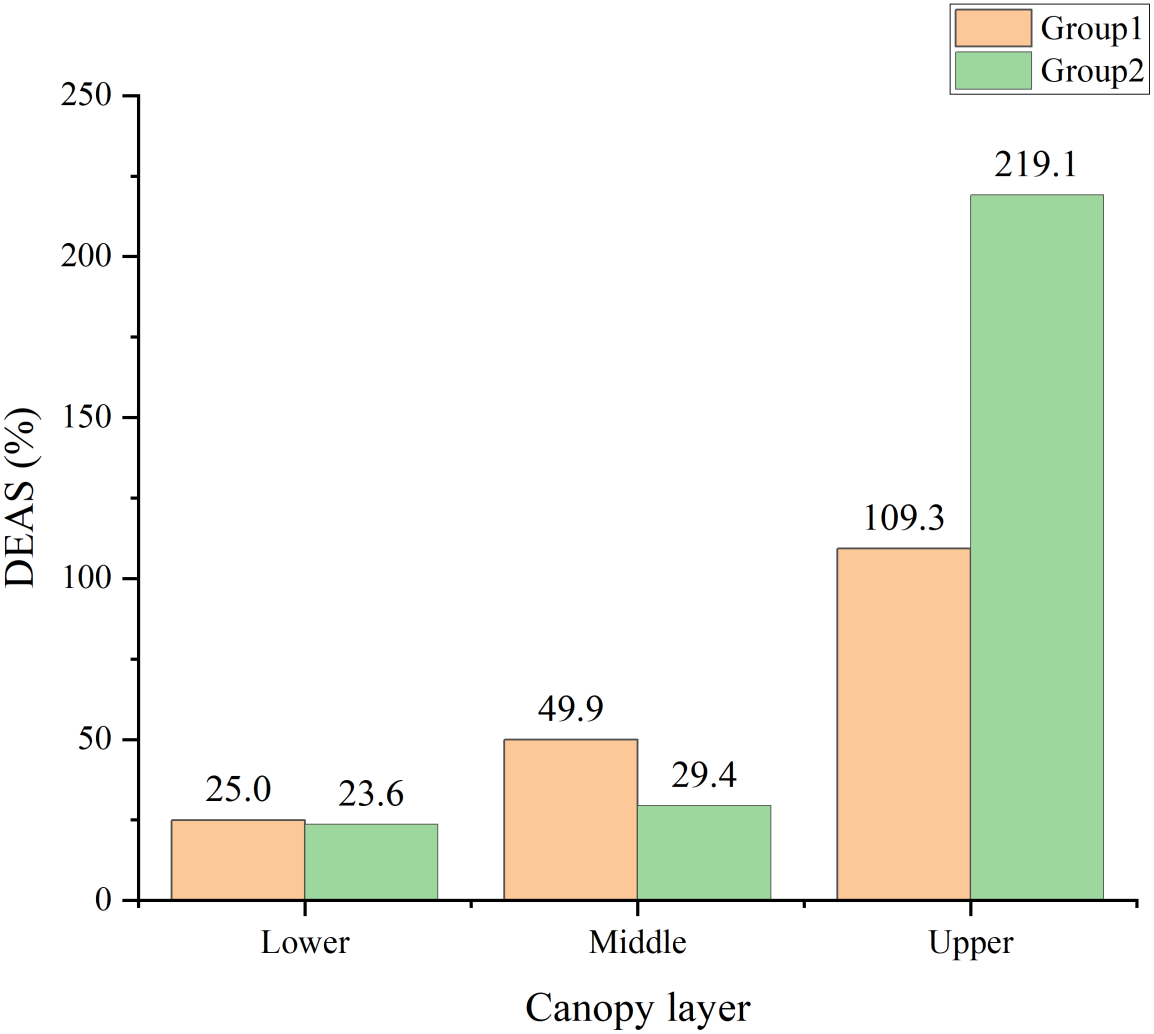
Spray deposition efficiency on the abaxial surface (DEAS) in different layers of the canopy under the two treatments.

Differences in the DEAS were observed (**Figure 7**). In the lower layer, the two treatments showed low DEAS (25.0% in group 1 and 23.6% in group 2). In the middle layer, group 1 had a DEAS of 49.9%, whereas the DEAS of group 2 was 29.4%. High DEAS values were observed in the upper canopy layer (more than 100% for both treatments).

**Figure 8:**
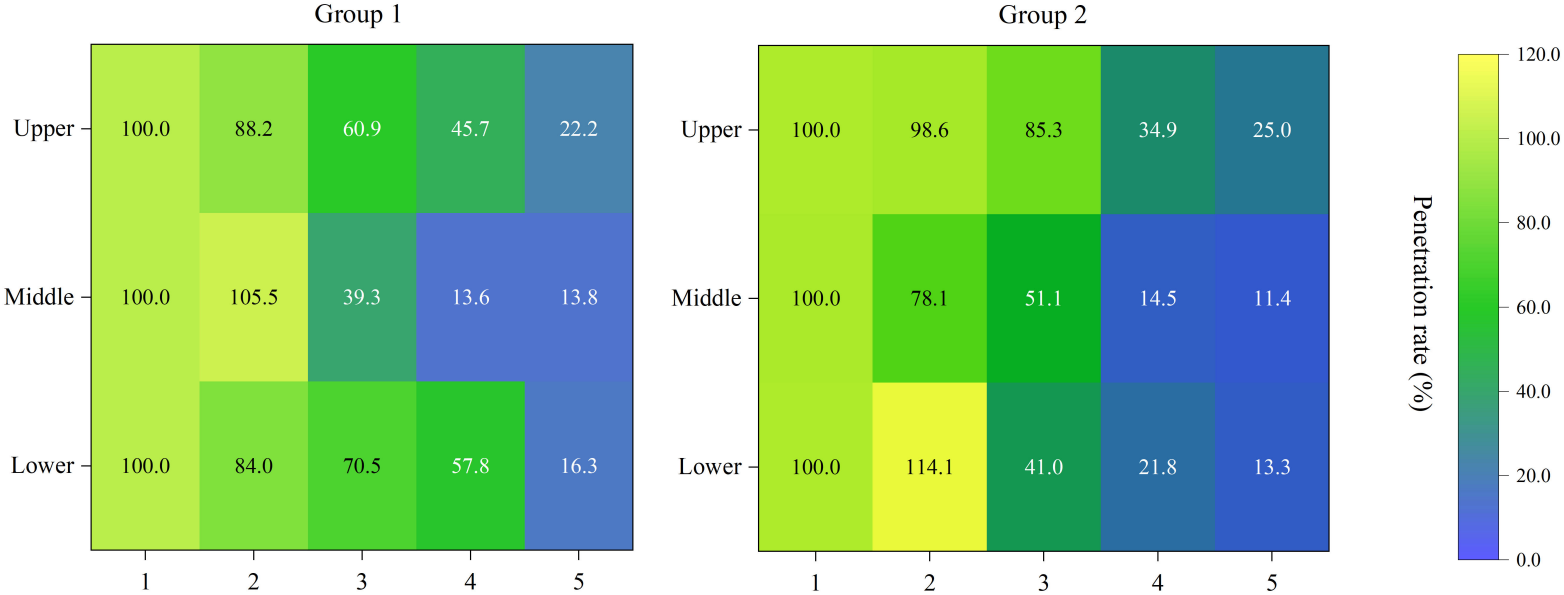
Spray penetration rate in different areas of the canopy under the two treatments.

The penetration rates in the different areas of the canopy are summarized in **Figure 8**. As seen in the figure, both treatments showed high penetration rates (>75%) in the proximal portion of the canopy (layers 1 and 2). In the center of the canopy (layer 3), the penetration rates were higher than 50% in most areas. Area M3 in group 1 and L3 in group 2 had relatively low penetration rates of approximately 40%. The penetration rates decreased severely in the distal portion of the canopy (layers 4 and 5). In the middle layer, the penetration rates for all areas were less than 15%.

## 4 Discussion

Spray deposition on the leaf abaxial surface has been the focus of increasing research attention in recent years (Maski and Durairaj, 2010; Owen-Smith et al., 2019; Jiang et al., 2023). Air-assisted spraying has been shown to achieve superior deposition on the abaxial surface of leaves in practical application (Christovam et al., 2010; Sinha et al., 2020; Xu et al., 2023). However, most previous studies have been conducted to test the performance of sprayers. The mechanism of deposition on the leaf’s abaxial surface and the optimization of sprayer systems for field application are poorly investigated. In the present study, the patterns and efficiency of spray coverage were examined. Assessment of the effects of different application parameters revealed that the included angle, rotation speed of the fan, and nozzle type significantly influenced spray coverage on the leaf’s abaxial surface, of which the included angle had the strongest effect (**Table 4**). When the target is situated leeward, deposition of droplets on the target is difficult. Although the airflow may move around a barrier to a certain extent (Greenspan, 2009), only a limited number of droplets will reach the abaxial surface of leaves in this manner. Therefore, it is necessary to investigate strategies to enhance the exposure of the abaxial surface of leaves to the wind field.

The effect of airflow on the leaf has been studied previously. Wu et al. (2021) defined two states of blade motion in the wind field. The critical wind speed when the leaf motion state changes is important. The wind deflection area of the leaf is affected by wind speed (Zhang et al., 2022). The reconfiguration, vibration, and wake characteristics of leaves were investigated by Jiang et al. (2021). With improvements in simulation technology, CFD has been used to simulate the motion and deformation of plant leaves in wind fields. The results suggest that the wind field angle of wind-delivered spray must be adjusted according to the stem and leaf angles (Yan et al., 2022). However, the relationship needs to be quantified by conducting additional research.

The present research explored the probability of exposure of the leaf’s abaxial surface for different values of α. Guidance for the adjustment of the airflow angle was provided by fitting a growth curve. Airflow direction can be changed by adjustment of the deflector angle for most orchard air-assisted sprayers, although the degree of adjustment is relatively limited owing to the structure of the fan. Given that the airflows are derived from one axial fan, the interaction between the wind fields after splitting is negligible. However, for multi-head fan sprayers, the interaction between the fan units also needs to be considered. As the airflow is generated by different fans, the wind field of the adjacent fan units may be affected if the angle is adjusted excessively. In such a case, spray deposition will be unpredictable. In addition, the adjustable angle of the fan unit is often limited because of its structure. The fan unit may not be adjustable to the optimal angle when the leaf angle is extreme. In the present research, an α value of 5–10° was ideal when conditions enabled a combination of these factors. Based on the fitted curve, the probability of abaxial surface exposure was higher than 95% within this range. An α value of 10° was used in the field validation in the present study.

In the field application, differences in α led to changes in spray coverage of the abaxial surface. It is worth noting that the DEAS of the upper canopy layer was higher than 100% in both treatments. This may be because the canopy of the citrus trees was spindle-shaped. The density of the canopy in the upper layer was lower, and thus the wind field and droplets would experience less resistance (Rossi et al., 1992). Therefore, the exposure of the abaxial surface in the upper layer was more similar to that of a single leaf. The α value was within a desirable range in both treatments (10° in group 1 and 15° in group 2). In terms of fog droplet penetration, it was found that an excellent deposition rate of the outer canopy may lead to a decrease in the deposition amount of the inner canopy (M3 in group 1 and L3 in group 2). This may be caused by excessive interception of droplets by the leaves of layers 1 and 2. Previous studies have shown that the penetration rate of droplets in the canopy is changed with the adjustment of the airflow angle (Pergher et al., 1997; Li et al., 2022). However, such change was not observed in the present field application. This may be associated with the canopy density of the citrus trees.

Many systems have been developed to guide the adjustment of sprayer parameters (Doruchowski et al., 2013; Doruchowski et al., 2014; Bahlol et al., 2020). Canopy features, such as tree row volume and leaf wall area, have been used to inform the adjustment of sprayer parameters (Sutton, 1988; Toews and Friessleben, 2012; Zhou et al., 2012; Miguez et al., 2019). However, such systems are aimed at improvement of pesticide utilization as well as reducing spray drift. There has been a lack of research attention on deposition on the abaxial surface of leaves. In the present study, the α value was used as a reference to guide the regulation of airflow. The only measurement required is that of the inclination angle of the leaf, which is easily determined for field application.

## 5 Conclusions

The angle α between the leaf and the airflow in an air-assisted spray system was observed to be an important factor affecting spray deposition on the abaxial leaf surface. Although a proportion of the droplets were deposited on the abaxial surface, the quantity was limited. To achieve improved abaxial surface deposition, the relationship between α and the abaxial leaf surface exposure probability was examined. The α value was positively correlated with the abaxial leaf surface exposure probability. The trend was in accordance with the fitted logistic growth curve. When the α value was greater than 5°, the probability of abaxial surface exposure was greater than 95%. The airflow angle adjustment was shown to be reliable in a field application. When the airflow angle was adjusted according to the theoretical value, spray coverage of the abaxial surface was significantly increased and higher DEAS was observed. In addition, the droplet penetration rate was not significantly affected by the adjustment of the airflow angle. These results provide a reference for adjustment of the wind field of air-assisted sprayers in field applications.

## Data availability statement

The original contributions presented in this study are included in the article/supplementary material. Further inquiries can be directed to the corresponding author.

## Author contributions

Conceptualization, JS. Methodology, JS and SX. Investigation, XW, YJ, and SX. Experimental platform construction, CL. Sources, JS. Data curation, XR and YZ. Writing—original draft preparation, SX. Writing—review and editing, SX, XW, and JS. Funding acquisition, JS. All authors contributed to the article and approved the submitted version.

## References

[B1] Guidelines on minimum requirements for agricul_tural pesticide application equipment. Available at: https://www.fao.org/3/Y2765E/Y2765E00.htm.

[B2] Bahlol H. Y. Chandel A. K. Hoheisel G. A. Khot L. R. (2020). The smart spray analytical system: developing understanding of output air-assist and spray patterns from orchard sprayers. Crop Prot. 127, 104977. doi: 10.1016/J.CROPRO.2019.104977

[B3] Burgio G. Marchesini E. Reggiani N. Montepaone G. Schiatti P. Sommaggio D. (2016). Habitat management of organic vineyard in northern Italy: the role of cover plants management on arthropod functional biodiversity. Bull. Entomol. Res. 106, 759–768. doi: 10.1017/S0007485316000493 27312132

[B4] Carr M. K. V. (2013). The water relations and irrigation requirements of macadamia (Macadamia SPP.): a review. Exp. Agric. 49, 74–90. doi: 10.1017/S0014479712000804

[B5] Carr M. K. V. (2014). THE water relations and irrigation requirements of papaya (CARICA PAPAYA l.): a review. Exp. Agric. 50, 270–283. doi: 10.1017/S0014479713000380

[B6] Celen I. H. (2008). Effect of angle of sprayer deflector on spray distribution in dwarf apple trees. J. Agron. 7, 206–208. doi: 10.3923/ja.2008.206.208

[B7] Christovam R.S. Raetano C. G. Aguiar H. O. Jr. Dal-Pogetto M. H. F. D. A. Prado E. P. Gimenes M. J.. (2010). Air-assistance in sleeve boom spray in the control of soybean rust. Bragantia 69, 231–238. doi: 10.1590/S0006-87052010000100029

[B8] Churchill A. C. L. (2011). Mycosphaerella fijiensis, the black leaf streak pathogen of banana: progress towards understanding pathogen biology and detection, disease development, and the challenges of control. Mol. Plant Pathol. 12, 307–328. doi: 10.1111/j.1364-3703.2010.00672.x 21453427 PMC6640443

[B9] Doruchowski G. Balsari P. Gil E. Marucco P. Roettele M. Wehmann H. J. (2014). Environmentally optimised sprayer (EOS)–a software application for comprehensive assessment of environmental safety features of sprayers. Sci. Total Environ. 482–483, 201–207. doi: 10.1016/J.SCITOTENV.2014.02.112 24651055

[B10] Doruchowski G. Roettele M. Herbst A. Balsari P. (2013). Drift evaluation tool to raise awareness and support training on the sustainable use of pesticides by drift mitigation. Comput. Electron. Agric. 97, 27–34. doi: 10.1016/J.COMPAG.2013.06.006

[B11] Duga A. T. Ruysen K. Dekeyser D. Nuyttens D. Bylemans D. Nicolai B. M.. (2015). Spray deposition profiles in pome fruit trees: effects of sprayer design, training system and tree canopy characteristics. Crop Prot. 67, 200–213. doi: 10.1016/j.cropro.2014.10.016

[B12] FAO. (2021). Guidelines on minimum requirements for agricul_tural pesticide application equipment[R]. Available at: https://www.fao.org/3/Y2765E/Y2765E00.htm

[B13] Garcerá C. Fonte A. Moltó E. Chueca P. (2017). Sustainable use of pesticide applications in citrus: a support tool for volume rate adjustment. Int. J. Environ. Res. Public Heal. 14, 715. doi: 10.3390/IJERPH14070715 PMC5551153 28665344

[B14] Garcerá C. Vicent A. Chueca P. (2020). Effect of spray volume, application timing and droplet size on spray distribution and control efficacy of different fungicides against circular leaf spot of persimmon caused by plurivorosphaerella nawae. Crop Prot. 130, 105072. doi: 10.1016/J.CROPRO.2019.105072

[B15] Greenspan D. (2009). Computer studies of molecular air flow around a circular cylinder. Comput. Math. Appl. 58, 414–421. doi: 10.1016/J.CAMWA.2006.12.105

[B16] Grella M. Marucco P. Zwertvaegher I. Gioelli F. Bozzer C. Biglia A.. (2022). The effect of fan setting, air-conveyor orientation and nozzle configuration on airblast sprayer efficiency: insights relevant to trellised vineyards. Crop Prot. 155. doi: 10.1016/j.cropro.2022.105921

[B17] Jiang H. Xin D. Zhang H. (2021). Wind-tunnel study of the aerodynamic characteristics and mechanical response of the leaves of betula platyphylla sukaczev. Biosyst. Eng. 207, 162–176. doi: 10.1016/j.biosystemseng.2021.05.004

[B18] Jiang Y. Yang Z. Xu X. Shen D. Jiang T. Xie B.. (2023). Wetting and deposition characteristics of air-assisted spray droplet on large broad-leaved crop canopy. Front. Plant Sci. 14. doi: 10.3389/fpls.2023.1079703 PMC9895840 36743480

[B19] Laurence A. British Museum (Natural History), L Halsey S. H. (1978). Whitefly of the world; a systematic catalogue of the aleyrodidae (Homoptera) with host plant and natural enemy data.

[B20] Li J. Li Z. Ma Y. Cui H. Yang Z. Lu H. (2021). Effects of leaf response velocity on spray deposition with an air-assisted orchard sprayer. Int. J. Agric. Biol. Eng. 14, 123–132. doi: 10.25165/j.ijabe.20211401.5435

[B21] Li T. Qi P. Wang Z. Xu S. Huang Z. Han L.. (2022). Evaluation of the effects of airflow distribution patterns on deposit coverage and spray penetration in multi-unit air-assisted sprayer. Agronomy 12, 944. doi: 10.3390/agronomy12040944

[B22] Maski D. Durairaj D. (2010). Effects of charging voltage, application speed, target height, and orientation upon charged spray deposition on leaf abaxial and adaxial surfaces. Crop Prot. 29, 134–141. doi: 10.1016/j.cropro.2009.10.006

[B23] Miguez M. Deleon R. Vicente G. Zoppolo R. (2019). “Real time tree row volume estimation for efficient application of phytosanitary products in fruit trees,” in 2019 IEEE International Symposium on Circuits and Systems (ISCAS) (IEEE), Sapporo, Japan. pp. 1–3. doi: 10.1109/ISCAS.2019.8702561

[B24] Owen-Smith P. Wise J. Grieshop M. J. (2019). Season long pest management efficacy and spray characteristics of a solid set canopy delivery system in high density apples. Insects 10, 193. doi: 10.3390/insects10070193 31261916 PMC6681383

[B25] Pai N. Salyani M. Sweeb R. D. (2009). Regulating airflow of orchard airblast sprayer based on tree foliage density. Trans. ASABE 52, 1423–1428. doi: 10.13031/2013.29122

[B26] Pergher G. Gubiani R. Tonetto G. (1997). Foliar deposition and pesticide losses from three air-assisted sprayers in a hedgerow vineyard. Crop Prot. 16, 25–33. doi: 10.1016/S0261-2194(96)00054-3

[B27] Rossi F. Facini O. Predieri S. Georgiadis T. (1992). Relationships between canopy structure and wind speed pattern in a peach tree. Acta Hortic. 313, 157–164. doi: 10.17660/ActaHortic.1992.313.18

[B28] Sinha R. Ranjan R. Khot L. R. Hoheisel G. A. Grieshop M. J. (2020). Comparison of within canopy deposition for a solid set canopy delivery system (SSCDS) and an axial–fan airblast sprayer in a vineyard. Crop Prot. 132, 105124. doi: 10.1016/J.CROPRO.2020.105124

[B29] Sutton T. B. (1988). Evaluation of the tree-Row-Volume model for full-season pesticide application on apples. Plant Dis. 72, 629. doi: 10.1094/PD-72-0629

[B30] Svensson S. A. Brazee R. D. Fox R. D. Williams K. A. (2003). Air jet velocities in and beyond apple trees from a two-fan cross-flow sprayer. Trans. ASAE 46, 611–621. doi: 10.13031/2013.13587

[B31] Toews R.-B. Friessleben R. (2012). Dose rate expression–need for harmonization and consequences of the leaf wall area approach. Erwerbs-Obstbau 54, 49–53. doi: 10.1007/s10341-012-0161-z

[B32] TOPPS-Prowadis Project (2014) Best management practices to reduce spray drift. Available at: http://www.topps-life.org/.

[B33] Toselli M. Scudellari D. Fernandez V. Abadia J. (2009). Foliar nutrition of fruit trees. Italus Hortus 16, 45–54.

[B34] Wang J. Qi L. Xia Q. (2015). CFD simulation and validation of trajectory and deposition behavior of droplets around target affected by air flow field in greenhouse. Trans. Chin. Soc Agric. Eng. 31, 46–53. doi: 10.11975/j.issn.1002-6819.2015.11.007

[B35] Washington J. R. Cruz J. Lopez F. Fajardo M. (1998). Infection studies of mycosphaerella fijiensis on banana and the control of black sigatoka with chlorothalonil. Plant Dis. 82, 1185–1190. doi: 10.1094/PDIS.1998.82.11.1185 30845404

[B36] Wu S. Liu J. Wang J. Hao D. Wang R. (2021). The motion of strawberry leaves in an air-assisted spray field and its influence on droplet deposition. Trans. ASABE 64, 83–93. doi: 10.13031/trans.14143

[B37] Xu S. Feng Y. Han L. Ran X. Zhong Y. Jin Y.. (2023). Evaluation of the wind field and deposition effect of a novel air-assisted strawberry sprayer. Agriculture 13, 230. doi: 10.3390/agriculture13020230

[B38] Yan C. Niu C. Ma S. Tan H. Xu L. (2022). CFD models as a tool to analyze the deformation behavior of grape leaves under an air-assisted sprayer. Comput. Electron. Agric. 198, 107112. doi: 10.1016/j.compag.2022.107112

[B39] Zhang C. Zhou H. Xu L. Ru Y. Ju H. Chen Q. (2022). Measurement of morphological changes of pear leaves in airflow based on high-speed photography. Front. Plant Sci. 13. doi: 10.3389/FPLS.2022.900427/BIBTEX PMC9685665 36438116

[B40] Zhou L. Ling Z. XinYu X. WeiMin D. Zhu S. QingQing Z.. (2016). Design and experiment of 3WQ-400 double air-assisted electrostatic orchard sprayer. Trans. Chin. Soc Agric. Eng. 32, 45–53. doi: 10.11975/j.issn.1002-6819.2016.16.007

[B41] Zhou J. He X. Landers A. J. (2012). Dosage adjustment for pesticide application in vineyards. Trans. ASABE 55, 2043–2049. doi: 10.13031/2013.42490

[B42] Zhu H. Derksen R. C. Krause C. R. Brazee R. D. Fox R. D. Ozkan H.E. (2004). Spray deposition in taxus and air velocity profile for a fiveport, air-assist sprayer. ASABE. doi: 10.13031/2013.17054

